# D-MGDCN-CLSTM: A Traffic Prediction Model Based on Multi-Graph Gated Convolution and Convolutional Long–Short-Term Memory

**DOI:** 10.3390/s25020561

**Published:** 2025-01-19

**Authors:** Linliang Zhang, Shuyun Xu, Shuo Li, Lihu Pan, Su Gong

**Affiliations:** 1 Shanxi Intelligent Transportation Laboratory Co., Ltd., Taiyuan 030036, China; 2 Shanxi Intelligent Transportation Institute Co., Ltd., Taiyuan 030036, China; 3 School of Computer Science and Technology, Taiyuan University of Science and Technology, Taiyuan 030024, China; 4 School of Information Science and Technology, Southwest Jiaotong University, Chengdu 611756, China

**Keywords:** intelligent transportation systems, traffic prediction, deep learning, graph convolutional networks, spatio-temporal feature

## Abstract

Real-time and accurate traffic forecasting aids in traffic planning and design and helps to alleviate congestion. Addressing the negative impacts of partial data loss in traffic forecasting, and the challenge of simultaneously capturing short-term fluctuations and long-term trends, this paper presents a traffic forecasting model, D-MGDCN-CLSTM, based on Multi-Graph Gated Dilated Convolution and Conv-LSTM. The model uses the DTWN algorithm to fill in missing data. To better capture the dual characteristics of short-term fluctuations and long-term trends in traffic, the model employs the DWT for multi-scale decomposition to obtain approximation and detail coefficients. The Conv-LSTM processes the approximation coefficients to capture the long-term characteristics of the time series, while the multiple layers of the MGDCN process the detail coefficients to capture short-term fluctuations. The outputs of the two branches are then merged to produce the forecast results. The model is tested against 10 algorithms using the PeMSD7(M) and PeMSD7(L) datasets, improving MAE, RMSE, and ACC by an average of 1.38% and 13.89%, 1% and 1.24%, and 5.92% and 1%, respectively. Ablation experiments, parameter impact analysis, and visual analysis all demonstrate the superiority of our decompositional approach in handling the dual characteristics of traffic data.

## 1. Introduction

Traffic prediction is an essential component of Intelligent Transportation Systems (ITSs) [1], aiming to forecast the future conditions of urban transportation systems, such as traffic flow, speed, and passenger demand. Its purpose is to optimize the allocation and scheduling of transportation resources, helping travelers to better plan their daily trips, while also alleviating traffic congestion and mitigating environmental issues.

Traffic prediction aims to forecast future traffic conditions based on historical traffic data and the structure of the traffic network. Traditional prediction methods include statistical approaches and machine learning, treating prediction as a regression problem. The most representative algorithms are the Historical Average (HA) and the Auto-Regressive Integrated Moving Average (ARIMA) [2]. However, these methods typically only consider temporal information and ignore the spatial correlations in traffic data. Later, some traditional machine learning methods were proposed to model more complex data, such as Vector Auto-Regressive (VAR) [3] models, to address traffic prediction problems. While these methods can model more complex data, they require high data quality, and still cannot handle non-linear and non-stationary time series data effectively. With the rise of deep learning, as seen in areas like speech recognition, natural language processing, and image processing, more researchers have applied deep learning to spatio-temporal data prediction tasks, developing various deep learning-based traffic prediction models [4,5,6]. These models can provide real-time road traffic information to drivers, improving road utilization.

However, due to the complex correlations between time and space, this task remains challenging. On the one hand, one limitation lies in data incompleteness. In real-world traffic data collection, factors such as sensor malfunctions and communication disruptions often lead to missing data [7,8]. If these missing data are not properly handled, the prediction models may make inferences based on incomplete data, thereby reducing the accuracy of the predictions. On the other hand, traffic time series data exhibit highly dynamic behavior, with patterns shaped by both short-term fluctuations (such as traffic peaks and sudden events) and long-term trends (such as daily commuting patterns and seasonal variations). If a model focuses only on one of these characteristics, it may overlook some significant factors, such as changes in traffic patterns caused by holidays or special events. Existing models often fail to consider both of these key characteristics simultaneously. Additionally, the non-Euclidean structure of traffic networks adds further complexity to analysis and prediction, as roads that are geographically close but have opposite directions of travel may exhibit vastly different traffic conditions.

In summary, to address the impact of missing data on prediction accuracy, and the limitations of existing models in simultaneously considering short-term fluctuations and long-term trends, this paper proposes the D-MGDCN-CLSTM traffic prediction model, based on Multi-Graph Gated Dilated Convolution and Conv-LSTM. The main contributions are as follows:

- A DTWN similarity-based imputation module is proposed. This module calculates the similarity between time series using Dynamic Time Warping (DTW) and fills in missing data by averaging the K most similar values, ensuring the completeness of the traffic data.
- Considering the two major characteristics of traffic data—short-term fluctuations and long-term trends—this paper uses the Discrete Wavelet Transform (DWT) to decompose traffic data into approximation coefficients and detail coefficients. The approximation coefficients capture the long-term trends in traffic flow, while the detail coefficients contain short-term fluctuation information. Different models are applied to handle the data obtained from the DWT.
- To accurately capture these two data characteristics, this paper designs the Multi-Graph Gated Dilated Convolution Network (MGDCN) to process the detail coefficients and capture short-term fluctuations, while Conv-LSTM is used to process the approximation coefficients and capture long-term trends. This decompositional modeling strategy not only improves the model’s ability to capture both short-term and long-term traffic data features, but also enables a more comprehensive understanding and prediction of complex variations in traffic flow.

## 2. Related Work

### 2.1. Research on Traffic Prediction Methods

The task of traffic prediction aims to forecast future traffic conditions using historical traffic data, which involves an in-depth analysis of the spatial structure of traffic networks and temporal characteristics, as well as understanding the spatio-temporal dependencies between different locations. To capture spatial features, researchers have utilized Convolutional Neural Networks (CNNs) to extract spatial features from traffic images or map data, combined with Recurrent Neural Networks (RNNs) and their variants to capture temporal features [9,10,11]. However, traffic networks are distributed in a non-Euclidean structure, making CNN-based models inadequate for accurately capturing the spatial relationships in traffic networks. In contrast, Graph Convolutional Networks (GCNs) [12] extend convolution operations to non-Euclidean spaces, and have gained attention for their advantages in handling such structures in traffic networks. Traditional methods use predefined adjacency matrices to capture spatial correlations, but these methods lack flexibility in adapting to the dynamic changes and complexities of road networks. In contrast, adaptive adjacency matrices dynamically adjust based on real-time traffic data, more accurately modeling actual traffic patterns and dependencies between roads. For example, the AGCRN [13] designs adaptive adjacency matrices and proposes a matrix decomposition strategy to break down weights into smaller matrices, dynamically capturing spatio-temporal dependencies without prior knowledge. Although adaptive adjacency matrices provide effective modeling for dynamic traffic networks, they may fail to accurately capture potential traffic pattern changes in the absence of sufficient historical data. As a result, many researchers have begun integrating different semantic graphs to more comprehensively capture complex spatio-temporal correlations. For instance, the DSTAGNN [14] constructs dynamic spatio-temporal awareness graphs based on the spatial correlation between historical traffic data, replacing predefined static graphs in traditional graph convolution, using multi-head attention mechanisms to adaptively adjust the spatial weights of nodes and combining multi-scale gated convolutions to further capture the dynamic spatio-temporal correlations of time series data. The STFGNN [15] captures the spatial characteristics of traffic networks by processing and integrating multiple spatio-temporal graphs in parallel, and it designs gated convolutions to learn temporal features. However, multi-graph approaches cannot fully meet the increasingly complex temporal dependencies and real-time changes in sequence data. Researchers have started developing prediction models using an encoder–decoder architecture. For example, the Multi-Scale Spatio-Temporal Aware Transformer (MSSTAT) model [16] combines a multi-scale transformer architecture with a data-driven graph convolution network, enabling it to capture static geographical features and dynamically reflect similarities in traffic patterns, thereby better simulating and predicting traffic flow. Nevertheless, existing models often overlook the two key characteristics of traffic data: short-term fluctuations and long-term trends, which can lead to a delayed or inaccurate response to traffic conditions, especially when unexpected events occur.

### 2.2. Research on Methods for Missing Data

In the process of traffic data collection, the issue of missing data is widespread. However, most existing prediction models tend to either ignore or assume complete data, simplifying the model development and computation process while neglecting the reality of incomplete data, which results in inaccurate predictions. Although some studies have attempted to fill in missing data using methods such as mean imputation, hot deck imputation, or random imputation [17,18], these approaches often fail to account for temporal dependencies, such as seasonal patterns and trend changes. Mean imputation may smooth out too much data detail, while hot deck imputation may introduce bias by selecting inappropriate “neighboring” data. Over time, more sophisticated statistical methods, such as regression imputation, K-nearest neighbors (KNN), and multiple imputation [19,20,21,22], have been widely adopted. KNN imputation works by finding the K nearest neighbors to the missing data point in terms of Euclidean distance, and using their values for imputation. Multiple imputation generates several possible complete datasets during the imputation process to estimate the uncertainty of the imputation. These methods better preserve the essential characteristics and structure of the data, providing more precise imputation than simpler methods. Lana et al. [23] combined multiple techniques, such as machine learning methods and heuristic optimization algorithms, to explore two strategies for missing data imputation. The SuperMICE model [24] integrates multiple imputation with machine learning algorithms to address missing data issues more comprehensively, effectively handling numerical gaps in data. However, existing imputation methods often overlook a critical factor: the similarity between data points. This leads to imputation processes that fail to fully exploit the intrinsic relationships and patterns within the data, preventing the imputation from truly reflecting the original nature of the data.

## 3. Model Design

### 3.1. Problem Definition

Traffic Network: The topological structure of a traffic road network is defined as an undirected graph G = (V, E, A), where V represents the set of sensor nodes in the road network, E represents the set of undirected edges between nodes in graph G, and A ∈ R^N×N^ corresponds to the adjacency matrix of the graph, with N being the number of sensor nodes. The graph nodes represent sensors on specific roads in the traffic network, which are responsible for recording traffic data on each road, such as traffic flow, vehicle speed, road occupancy rate, etc.

Traffic Features: The traffic information on the road network is defined as the attribute features of the nodes $X=\left(X_{t-T+1},X_{t-T+2},\ldots ,X_{t}\right)\in R^{N\times T}$, where N represents the number of nodes, and T represents the number of node attributes (the length of the historical time series). $X_{t}$ denotes the traffic feature values of all the nodes at time step t.

Therefore, the spatio-temporal traffic prediction problem can be viewed as learning a mapping function f(·) from given historical traffic data X and the topological structure of the traffic network G, to predict the traffic data for all nodes over the next p time steps, i.e., $\left\{X_{t+1},X_{t+2},\ldots ,X_{t+p}\right\}$. The mapping relationship can be represented as follows:

(1) $$\left\{X_{t+1},X_{t+2},\ldots ,X_{t+p}\right\}=f\left(\left\{X_{t-T+1},X_{t-T+2},\ldots ,X_{t}\right\};G\right),$$

### 3.2. Framework

**Figure 1:**
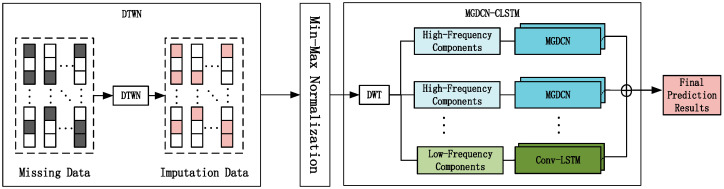
The overall framework of the D-MGDCN-CLSTM model.

The overall framework of the proposed D-MGDCN-CLSTM model is shown in Figure 1. It consists of the DTWN similarity-based imputation module and the MGDCN-CLSTM, a multi-graph gated long–short-term memory convolutional network module. First, the DTWN module is used to preprocess incomplete data. It calculates the similarity between time series using the Dynamic Time Warping (DTW) method and fills in the missing data by averaging the top K most similar values. Then, Min–Max normalization is applied to ensure data uniformity.

Next, the data are input into the MGDCN-CLSTM module to capture spatio-temporal features. The time series data are first processed by the DWT module, which performs multi-scale analysis on the raw traffic data to obtain two components: approximation coefficients and detail coefficients, in order to capture the two key features of traffic data—short-term fluctuations and overall trends. The detail coefficients are fed into the Multi-Graph Gated Convolution Network (MGDCN) to capture short-term fluctuations. This module includes the Multi-Graph Convolution Network (MGCN) for capturing spatial features with different semantic meanings in the traffic network, and the Gated Dilated Convolution Network (GDCN) for capturing temporal features. The approximation coefficients are input into the Conv-LSTM module to capture long-term trend features. Finally, the features from the two components are fused to produce the final prediction results.

The following sections will provide a detailed description of each module in the D-MGDCN-CLSTM model, explaining how they contribute to an overall improvement in prediction performance.

### 3.3. DTWN Module

Due to external factors that are beyond control, such as sensor failures, traffic data may have missing portions, making it difficult to accurately capture the actual changes in traffic flow, thus reducing prediction accuracy. KNN imputation is a commonly used method for filling in missing data, but filling in missing values based on Euclidean distance has certain limitations. For example, a road may have completely different traffic flows and behavior patterns depending on the direction of travel, so Euclidean distance cannot accurately reflect the similarity between traffic nodes.

To solve this issue, and considering that traffic data often exhibit temporal irregularities (such as differences in traffic patterns between different roads, weekdays, and weekends), this paper designs the DTWN module. Using the Dynamic Time Warping (DTW) algorithm to calculate the similarity between missing data points and other time series can more accurately capture the pattern-matching relationships of critical periods, such as peaks and troughs, in traffic sequences. DTW is a widely used algorithm in time series analysis, designed to measure the similarity between two sequences, even if they are nonlinearly aligned on the time axis. Its mathematical formula is as follows:

(2) $$D(i,j)=|x_{i}-y_{j}|+\min(D(i-1,j),D(i,j-1),D(i-1,j-1)),$$

Here, X = (x1, x2, …, xn) and Y = (y1, y2, …, yn) represent the traffic sequence data for nodes i and j, respectively. The first term, |*xi* − *yj*|, measures the difference between nodes *xi* and *yj*, while the second term, min(*D*(*i* − 1, *j*), *D*(*i*, *j* − 1), *D*(*i* − 1, *j* − 1)), represents the minimum cumulative cost of the path. By solving this recursively, the optimal alignment path between the two sequences is obtained. Using this approach, DTW effectively handles the nonlinear time alignment characteristics in traffic sequences, resulting in a similarity value *D*(*i*, *j*) for nodes i and j. A higher value indicates greater similarity in shape and patterns between the two time series. Based on the similarity values of each missing data point, the K most similar time series *υ*k are identified. The weighted average of these most similar series points is then used as the imputed value, reducing the impact of a single similarity value on the interpolation result. The imputed data point is inserted into the original sequence X to obtain the completed sequence data. The mathematical expression for this is as follows:

(3) $$x_{\mathrm{imputed}}=\frac{1}{K}\sum_{k\in K_{\mathrm{nearest}}}v_{k},$$

X′ = (*x*1, *x*2, …, *x*i − 1, *x*imputed, *x*i + 1, …, *x*n), (4)

Here, *vk* represents the values of the similar points, *K*nearest refers to the set of the *K* most similar data points, *x*imputed denotes the imputed data value, and X′ represents the sequence after the missing values are filled in. The algorithm is described in Algorithm 1. By using the DTWN module to handle the issue of missing traffic data, the imputation process not only considers the similarity between data points, but also reduces the influence of any single similarity value on the results. This approach provides a stable and accurate data foundation for the decomposed MGDCN-CLSTM traffic prediction model.
**Algorithm 1: DTWN for missing data imputation****Input:** Incomplete traffic data X, number of similar series *K*_nearest_**Output:** Preprocessed traffic data X′for each data point x_i_ in X doCompute DTW similarity between missing time series x_i_ and other time series:D(i, j) = |x_i_ − x_j_| + min(D(i − 1, j), D(i, j − 1), D(i − 1, j − 1))Select top K most similar time series *υ*_k_ (k∈*K*_nearest_)Compute imputed value for missing data point:*x*_imputed_ = (1/K) * (∑k∈*K*_nearest_ *υ*_k_)Replace missing data point x_i_ in X with *x*_imputed_end forReturn the updated time series X′

### 3.4. MGDCN-CLSTM Module

The characteristics of traffic data mainly include short-term fluctuations (such as traffic flow variations caused by sudden events or temporary road closures) and long-term trends (such as cyclical changes during daily peak and off-peak hours). However, existing traffic prediction models do not differentiate between short-term fluctuations and long-term trends in traffic data, which leads to decreased prediction accuracy when dealing with complex traffic dynamics. To address this issue, we designed a decompositional MGDCN-CLSTM module to capture different features. The pseudocode of the algorithm is shown in Algorithm 2. This module consists of three components: the Discrete Wavelet Transform (DWT), the Multi-Graph Gated Convolutional Network (MGDCN), and the Convolutional Long–Short-Term Memory Network (CLSTM). The detailed structure of each module is explained in the following three sections.
**Algorithm 2: MGDCN-CLSTM for Spatio-Temporal Feature Extraction****Input:** Preprocessed traffic data X′**Output:** Predicted traffic data Y_pred_Decompose X′ into detail coefficients X_D_ and approximation coefficients X_A_:(X_A_, X_D_) = DWT(X′)// Short-term Trend Modeling with MGDCNCompute weighted adjacency matrices:A^s^_ij_ = α * A_person_ + β * A_spearson_; A_ij_ = exp(−d^2^_ij_/σ^2^), if exp(−d^2^_ij_/σ^2^)) ≥ εFor each layer *l* from 1 to *L*:Compute spatial features for *l*-th layer:H^l+1^ = σ(D~^−1/2^A~^−1/2^ D~^−1/2^H^(l)^W^(l)^)H_s_^l+1^ = σ(D_s_~^−1/2^A_s_~^−1/2^ D_s_~^−1/2^A_s_~H_s_^(l)^W_s_^(l)^)end forFuse multi-semantic spatial features:Hgcn = H^l+1^ + H_s_^l+1^// Capture temporal features using Dilated convolution:H_DCN_ = σ(DilationConv(H_gcn_))// Fuse spatial temporal featuresHfinal = Z * Hgcn + (1- − Z)HDCN// Long-term Trend Modeling with Conv-LSTMfor each time step t do:Input X_A_ to Conv-LSTM:F_long_ = ConvLSTM(X_A_)end for// Feature Fusion:Y_pred_ = F_short_ + F_long_return Y_pred_

#### 3.4.1. DWT Module

The wavelet transform is widely used in the field of signal processing for feature extraction in both the time and frequency domains of non-stationary signals. Its multi-resolution analysis capability makes it a powerful tool for handling complex time series data. In this paper, we use the Discrete Wavelet Transform (DWT) to input the time series into a low-pass filter and a high-pass filter, resulting in different components: approximation coefficients dA and detail coefficients dD. Specifically, this process can be represented by the following equations:

(5) $$dA_{j+1}=\sum_{n}L(n-2k)x_{j},$$

(6) $$dD_{j+1}=\sum_{n}H(n-2k)x_{j},$$

**Figure 2:**
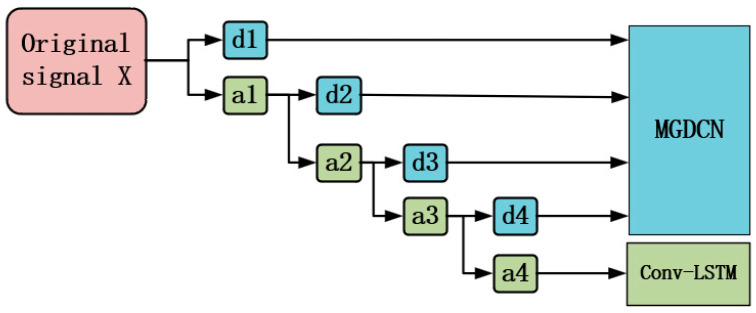
Diagram of DWT decomposition.

In this case, x represents the original data series. The low-pass filter L(n) is used to capture the overall trend and major changes in the signal, with the output being the approximation coefficients dA. The high-pass filter H(n) is used to capture the fluctuations or detailed differences in the signal, with the output being the detail coefficients dD. The DWT decomposition process is shown in Figure 2. First, the original time series data are input into low-pass and high-pass filters to obtain the first-level approximation coefficient dA1 and detail coefficient dD1. Then, the approximation coefficient is input into the low-pass and high-pass filters again to obtain the second-level approximation coefficient dA2 and detail coefficient dD2. This process is iteratively repeated, continuously extracting different frequency components of the signal, until the desired decomposition level is reached. To more clearly capture the variations in traffic data across different time scales, we designed two modules, MGDCN and Conv-LSTM, to handle the detail coefficients dD and the approximation coefficients dA, respectively. These modules not only enhance the model’s ability to adapt to changes at different time scales, but also allow for more accurate reflection and prediction of complex traffic flow dynamics.

#### 3.4.2. MGDCN

**Figure 3:**
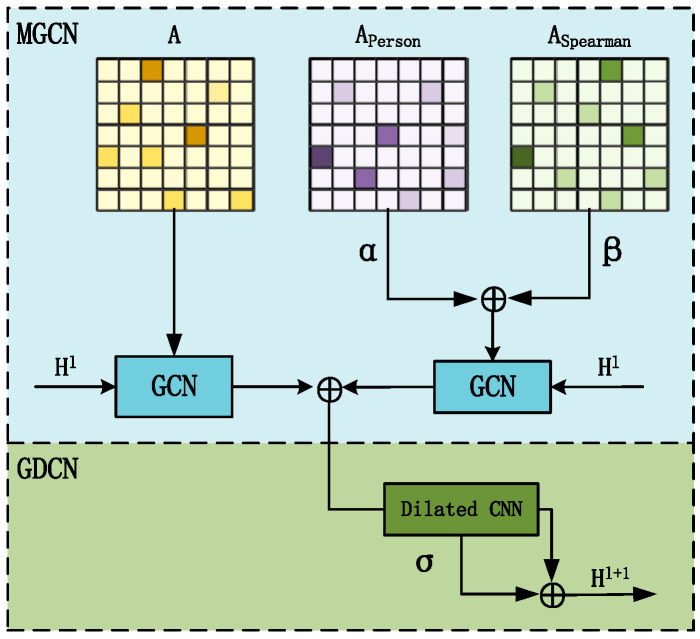
MGDCN structure.

To handle the detail coefficient components, we designed the Multi-Graph Gated Convolutional Network (MGDCN) module, as shown in Figure 3. The MGDCN consists of two parts: the Multi-Graph Convolutional Network (MGCN) and the Gated Dilated Convolutional Network (GDCN). The MGCN focuses on capturing the spatial features of different semantics in the traffic network by integrating a predefined adjacency matrix with similarity matrices based on various similarity measures. The weighted parameters *α* and *β* are used to adjust the matrices Aperson and Aspearson, allowing the model to adapt to the dynamic spatial relationships in the traffic flow. The GDCN, on the other hand, captures temporal features through a gating mechanism and dilated convolution. The dilated receptive field of the dilated convolution allows the network to capture a broader neighborhood of nodes. The activation function *σ* is applied to the output of the dilated convolution, enabling the model to learn more complex feature representations. Finally, the activated output is combined with the previous layer’s hidden state H^1^ through an addition operation, producing the next layer’s hidden state H^1+1^.

The MGCN module designs two different adjacency matrices: the predefined adjacency matrix A and the dynamic similarity matrix A^s^, which are input into the GCN to capture the different semantic spatial features of the traffic network. The formulas are as follows:

(7) $$A_{ij}=\left\{\begin{array}{c} \exp\left(-\frac{d_{ij}^{2}}{\sigma^{2}}\right),\mathrm{if}\exp\left(-\frac{d_{ij}^{2}}{\sigma^{2}}\right)\geq \varepsilon \\ 0\;\;,\;\mathrm{otherwise} \end{array}\right.,$$

(8) $$\begin{array}{l} {A^{s}}_{i,j}=\alpha *Person(X^{i},Y^{j})+\beta *Spearman(X^{i},Y^{j})\\ =\alpha \cdot \left(\frac{\sum_{k=1}^{n}\left(X_{k}^{i}-\bar{X^{i}}\right)(Y_{k}^{j}-\bar{Y^{j}})}{\sqrt{\sum_{k=1}^{n}{\left(X_{k}^{i}-\bar{X^{i}}\right)}^{2}}\sqrt{\sum_{k=1}^{n}{\left(Y_{k}^{j}-\bar{Y^{j}}\right)}^{2}}}\right)+\beta \cdot \left(1-\frac{6\sum_{k=1}^{n}d_{k}^{2}}{n(n^{2}-1)}\right) \end{array},$$

where X^i^ = (x_1_, x_2_, …, x_n_) and Y^j^ = (y_1_, y_2_, …, y_n_) represent the traffic time series data of nodes i and j, respectively. $Person(X^{i},Y^{j})$ and $Spearman(X^{i},Y^{j})$ represent the Pearson and Spearman correlation coefficients between series $X^{i}$ and $Y^{j}$, respectively. *α* and *β* are the weighting factors controlling the influence of these two coefficients, and *ε* is the standard deviation of the distance. Given the adjacency matrix A and similarity matrix As, we used the GCN to capture the spatial features of the traffic network under different semantic contexts. The formulas are as follows:

(9) $$H^{l+1}=\sigma ({\tilde{D}}^{-1/2}{\tilde{A}}^{-1/2}{\tilde{D}}^{-1/2}H^{l}W^{l}),$$

(10) $$H_{s}^{l+1}=\sigma ({{\tilde{D}}_{s}}^{-1/2}{{\tilde{A}}_{s}}^{-1/2}{{\tilde{D}}_{s}}^{-1/2}{H_{s}}^{l}{W_{s}}^{l}),$$

where σ(·) represents the nonlinear activation function, $W^{l}$ and ${W_{s}}^{l}$ denote the weight matrices of the l-th layer of the GCN, and ${\tilde{D}}^{-1/2}{\tilde{A}}^{-1/2}{\tilde{D}}^{-1/2}$ and ${{\tilde{D}}_{s}}^{-1/2}{{\tilde{A}}_{s}}^{-1/2}{{\tilde{D}}_{s}}^{-1/2}$ are the symmetric normalized matrices for the predefined adjacency matrix A and the similarity matrix A_s_, respectively. $\tilde{A}=A+I$, ${\tilde{A}}_{s}=A_{s}+I$, I represents the identity matrix, and $\tilde{D},{\tilde{D}}_{s}$ is the degree matrix for $\tilde{A},{\tilde{A}}_{s}$.The output result $H_{gcn}$ is obtained by merging the spatial features from different semantics, as shown in the following formula:

(11) $$H_{gcn}=H^{l+1}+{H_{s}}^{l+1},$$

**Figure 4:**
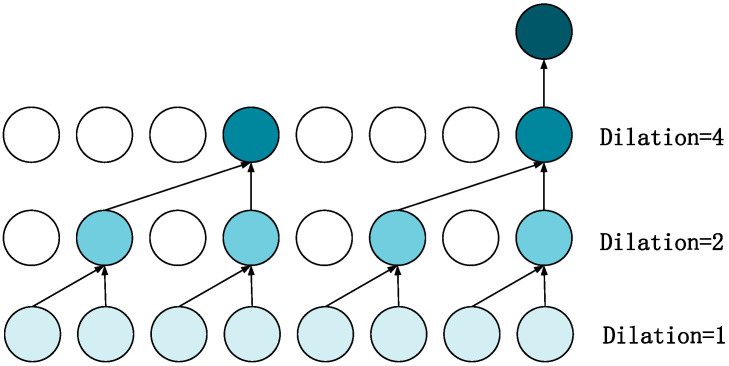
Dilated CNN structure.

The core of the GDCN is the Dilated Convolutional Neural Network (Dilated CNN), which increases the receptive field of the convolutional kernel through dilation. This allows the network to capture a broader range of contextual information without increasing computational complexity, thereby better modeling traffic flow characteristics across different time scales. Its structure is shown in Figure 4. In this structure, the GDCN first receives the output $H_{gcn}$ from the multi-graph convolution. The dilated convolution is used to capture long-term sequential features more effectively, and the gating mechanism is applied to combine these features, resulting in the spatio-temporal features $H_{final}$. The formula is as follows, where W_gcn_, W_DCN_, and b_z_ represent learnable parameters:

(12) $$H_{DCN}=\sigma \left(DilationConv\left(H_{gcn}\right)\right)$$

(13) $$H_{final}=Z*H_{gcn}+\left(1-Z\right)H_{DCN}$$

(14) $$Z=sigmoid\left(H_{gcn}W_{gcn}+H_{DCN}W_{DCN}+b_{z}\right)$$

#### 3.4.3. Conv-LSTM

**Figure 5:**
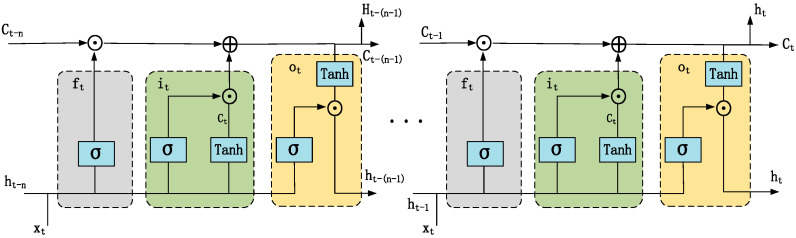
Conv-LSTM structure.

LSTM, which is widely used for capturing long-term features in time series, effectively addresses the vanishing gradient problem in traditional RNNs when handling long sequential data by using input gates, forget gates, and output gates to control the flow of information. However, it struggles to capture and utilize the continuity of spatial information. To address this issue, Convolutional LSTM (Conv-LSTM) was introduced. In Conv-LSTM, all input, forget, and output gates, as well as cell state updates, are performed through convolutional operations, allowing the network to retain the continuity of spatial information within time series data. Its structure is shown in Figure 5. The forget gate $f_{t}$ determines which feature information to retain, while the input gate $i_{t}$ controls the flow of feature information. The formulas for each gate are as follows:

(15) $$f_{t}=\sigma (W_{f}*[h_{t-1},x_{t}]+b_{f}),$$

(16) $$i_{t}=\sigma (W_{i}*[h_{t-1},x_{t}]+b_{i}),$$

(17) $${\tilde{C}}_{t}=\tanh(W_{C}*[h_{t-1},x_{t}]+b_{C}),$$

where $f_{t}$, $i_{t}$ and ${\tilde{C}}_{t}$ represent the forget gate, input gate, and hidden state at the current time step, respectively. $W_{f}$, $W_{i}$, $W_{C}$ and $b_{f}$, $b_{i}$, $b_{C}$ are the trainable parameters for each gate. σ(·) denotes the sigmoid activation function, and * represents the convolution operation. After the forget and input gates are calculated, the candidate cell state is merged with the current hidden state ${\tilde{C}}_{t}$ and the previous hidden state $C_{t-1}$. The formula is as follows:

(18) $$C_{t}=f_{t}⊙C_{t-1}+i_{t}⊙{\tilde{C}}_{t},$$

where Wc and $b_{c}$ are the trainable weights, and ${\tilde{C}}_{t}$ and $C_{t}$ represent the cell state at time t and its updated value, respectively. ⊙ denotes the Hadamard product (element-wise multiplication). After the cell state is updated, the output gate $o_{t}$ determines which information from the cell state is output. The output is then element-wise multiplied with the cell state, which is passed through the tanh activation function to produce the final result $h_{t}$. The formulas are as follows:

(19) $$o_{t}=\sigma (W_{o}*[h_{t-1},x_{t}]+b_{o}),$$

(20) $$h_{t}=o_{t}⊙\tanh(C_{t}),$$

where $h_{t}$ represents the output state, and $W_{o}$ and $b_{o}$ are the trainable weights.

## 4. Experiments

### 4.1. Dataset

To validate the predictive performance of the D-MGDCN-CLSTM model, a series of experiments were conducted using the PeMSD7(M) and PeMSD7(L) datasets. These datasets were collected in real time by the California Performance Measurement System (PEMS) at 30 s intervals. The datasets cover traffic information collected on weekdays from May to June 2012, aggregated into 5 min time intervals. The PeMSD7(M) dataset contains data from 228 sensors, while the PeMSD7(L) dataset includes data from 1026 sensors. Detailed information can be found in Table 1. In this study, the datasets were divided into training, validation, and testing sets in a 7:2:1 ratio, based on time order. The model uses the past 10 consecutive time steps to predict traffic conditions for the next 15 min, 30 min, and 60 min.

### 4.2. Evaluation Metircs

To evaluate the performance of the model, three evaluation metrics were used: Mean Absolute Error (MAE), Root Mean Squared Error (RMSE), and Accuracy (ACC). A higher Accuracy value indicates a better predictive capability of the model. The formulas for these metrics are as follows:

(21) $$MAE\left(Y,\hat{Y}\right)=\frac{1}{N}\sum_{i=1}^{N}\left|Y_{i}-{\hat{Y}}_{i}\right|,$$

(22) $$RMSE\left(Y,\hat{Y}\right)=\sqrt{\frac{1}{N}\sum_{i=1}^{N}{\left(Y_{i}-{\hat{Y}}_{i}\right)}^{2}},$$

(23) $$Accuracy=1-\frac{{\left\|Y-{\hat{Y}}_{i}\right\|}_{F}}{{\left\|Y_{i}\right\|}_{F}},$$

where N is the total number of nodes, $Y_{i}$ is the true value, and ${\hat{Y}}_{i}$ is the predicted value.

### 4.3. Experimental Setup

The experiments in this paper were conducted using a model built in an environment based on Python 3.9, PyTorch 2.2.0, and CUDA 11.5. The hardware configuration included an Intel(R) Xeon(R) Gold 6330 CPU @ 2.00GHz, an NVIDIA RTX 3090 GPU, with a system disk size of 30 GB and 14 GB of GPU memory. A sliding time window approach was adopted, using the past 10 time steps to predict traffic conditions for the next 15 min, 30 min, and 60 min. Additionally, all traffic speed data were normalized to scale the values to the range [0, 1]. The Adam optimizer was used, with an initial learning rate of 0.001 and a batch size of 64. An early stopping strategy was introduced, with training terminated when performance on the validation set showed no improvement for 15 consecutive epochs. The main hyperparameters during the model training phase included the number of hidden units, the number of stacked layers, and the number of DWT decomposition levels. For the PEMSD7(M) dataset, the number of hidden units was set to 128, the number of stacked layers to 3, and the DWT decomposition levels to 3. For the PEMSD7(L) dataset, the number of hidden units was set to 128, the number of stacked layers to 2, and the DWT decomposition levels to 4. These parameter settings were tailored to the scale and characteristics of different datasets to ensure that the model effectively captured spatio-temporal features within various network structures, enhancing predictive performance.

### 4.4. Comparative Experimental Analysis

To evaluate the predictive performance of the D-MGDCN-CLSTM model, the following existing prediction models were selected for comparison: the Graph Convolutional Network (GCN) [12], the Spatio-Temporal Synchronous Graph Convolutional Network (STSGCN) [25], Temporal Graph Convolution (T-GCN) [26], Spatio-Temporal Graph Convolution (STGCN) [27], the Diffusion Convolutional Recurrent Neural Network (DCRNN) [28], Graph-WaveNet [29], the Spatio-Temporal Attention Graph Convolutional Network (ASTGCN) [30], the Long Short-Term Graph Convolutional Network (LSGCN) [31], the Spatio-Temporal Graph ODE Network (STGODE) [32], and the Spatio-Temporal Fusion Graph Neural Network (STFGNN) [15].

The average metrics of the different models at various prediction time intervals are shown in Table 2. It can be observed that compared to the GCN model, the D-MGDCN-CLSTM model improved the MAE and RMSE by 65.87% and 41.16%, and 73.18% and 53.24%, respectively, while the Accuracy (ACC) was improved by 12% and 11%. These improvements were mainly due to the GCN model’s ability to handle the graph structure of the traffic network, but its neglect of time series features. The T-GCN introduces GRU to ensure that the model can capture both spatial and temporal features. The STGCN, Graph WaveNet, and the LSGCN all combine 1D convolution (1D CNN) with gating mechanisms to capture temporal correlations. However, each of these models has certain limitations. The STGCN uses traditional Chebyshev graph convolution to extract spatial features, but relies on fixed polynomial kernels, limiting its adaptability to complex and dynamic traffic networks. In contrast, the D-MGDCN-CLSTM model addresses this by introducing a multi-scale analysis module and adaptive multi-graph convolution design, improving the MAE and RMSE by 25.9% and 26.9%, and 18.3% and 19.0%, and boosting the ACC by 10% and 4%. Graph WaveNet, with its adaptive adjacency matrix and diffusion convolution, excels at capturing dynamically changing graph structures. However, its ability to extract complex multi-semantic spatial features is limited. The D-MGDCN-CLSTM model enhances this through multi-graph convolution and gated dilated convolution modules, achieving MAE and RMSE improvements of 10.3% and 20.5%, and 15.2% and 22%, and ACC gains of 2% and 1%. The LSGCN uses a cosAtt module, based on graph attention, combined with graph convolution networks to extract node relationships and importance. However, it models temporal features in a simplistic way, failing to capture multi-scale temporal characteristics effectively. The D-MGDCN-CLSTM model leverages wavelet transforms, multi-graph convolution, and Conv-LSTM modules to capture both short-term fluctuations and long-term trends, improving the MAE and RMSE by 6.2% and 17.1%, and 8.8% and 15.6%, and the ACC by 4% and 5%. Similar improvements in MAE, RMSE, and ACC were observed on the PEMSD7(L) dataset, confirming its excellent performance in dynamic graph modeling, multi-semantic feature extraction, and temporal feature capture.

The DCRNN model relies on diffusion graph convolution and gated recurrent units (GRU) to capture spatio-temporal correlations. However, its diffusion convolution focuses only on local features, lacking the ability to capture global and multi-semantic features. Additionally, its GRU-based temporal modeling is suitable only for simple linear patterns. The D-MGDCN-CLSTM model addresses these issues, with MAE and RMSE improvements of 22.4% and 25.7%, and 25.3% and 30.9%, and ACC gains of 1% and 2%. The STFGNN considers global and local correlations by incorporating relationships between nodes with similar patterns. However, its node similarity evaluation has limited generalization, making it less effective in handling missing data and capturing short-term dynamics. The D-MGDCN-CLSTM model overcomes these shortcomings, enhancing the MAE and RMSE by 1.4% and 14.3%, and 1.2% and 6.4%, and boosting the ACC by 1%, demonstrating superior spatio-temporal modeling and prediction capabilities.

GCN-based models excel in processing graph-structured data, but rely on discrete operations, potentially losing information when simulating continuous dynamic processes. The STGODE replaces graph convolution with an ODESolver module to simulate continuous dynamic behaviors, enabling it to handle spatial and temporal continuity more naturally. However, it mainly focuses on independent temporal and spatial dynamics, overlooking their complex interactions. The D-MGDCN-CLSTM model addresses this, achieving MAE and RMSE improvements of 3.7% and 12.4%, and 7.0% and 7.5%, and ACC gains of 1%. The STFGNN fuses spatial and temporal features using spatio-temporal synchronous graphs, addressing the limitations of separate modeling. However, it fails to capture the spatial relationships between short-term fluctuations and long-term trends. The D-MGDCN-CLSTM model compensates for this by using multi-scale decomposition to capture both short- and long-term features, and combines multi-graph convolution with a dynamic filling mechanism, improving the MAE and RMSE by 3.7% and 14.3%, and 1.3% and 6.4%, and the ACC by 1%.

In summary, the D-MGDCN-CLSTM model demonstrates significant advantages in multi-scale spatio-temporal modeling, global and local feature extraction, and handling of missing data. Compared to various mainstream models, it achieves substantial improvements in MAE, RMSE, and ACC, validating its exceptional spatio-temporal prediction capabilities and robustness. This highlights the effectiveness of modeling spatio-temporal dependencies in a decomposed manner.

### 4.5. Parameter Impact Analysis

**Figure 6:**
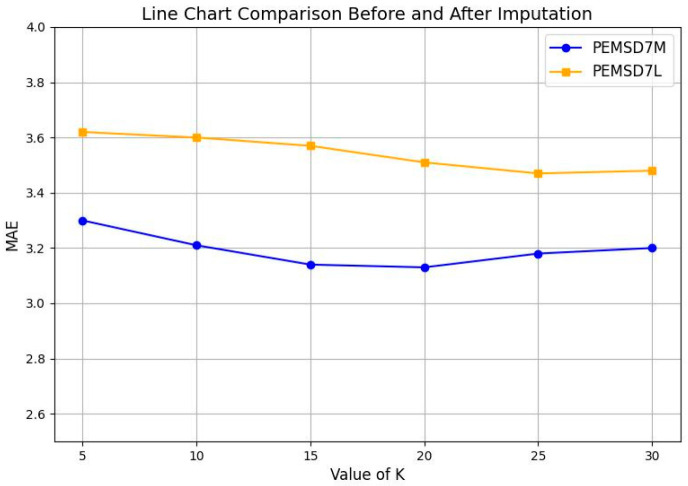
Experimental results of distortion rate before and after interpolation.

The choice of K in the DTWN module determines the balance between capturing local patterns and global trends in the interpolation method. A smaller K value emphasizes local patterns, while a larger K value results in smoother outputs, impacting the accuracy of the interpolation results. To evaluate the effect of different K values on interpolation performance, we randomly introduced a 10% missing rate in two datasets and adjusted K to observe its impact on the results. The mean absolute error (MAE) was selected to measure the difference between the interpolated data and the original data. As shown in Figure 6, the MAE exhibits a trend of first decreasing and then increasing with changes in K. For the PEMSD7(M) dataset, the MAE reaches its minimum when K = 20, while for the PEMSD7(L) dataset, the minimum MAE occurs at K = 25. This indicates that a moderate K value can balance local and global features, improving the interpolation performance. These findings provide a reliable basis for parameter selection in subsequent research.

**Figure 7:**
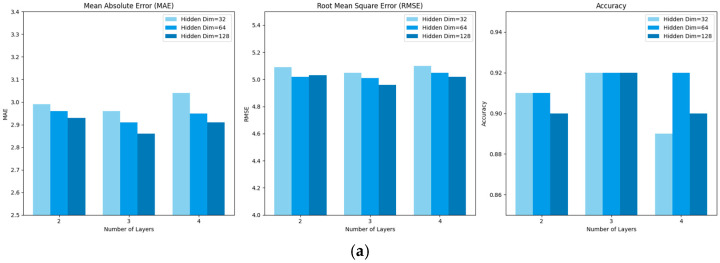

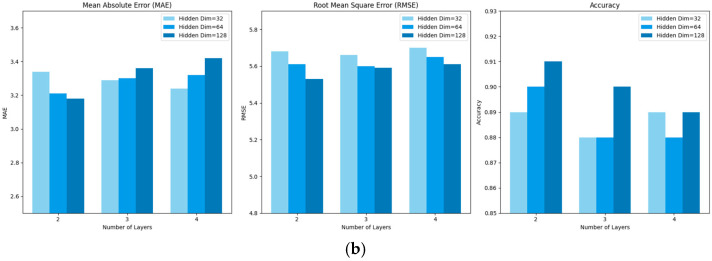
Experimental results with different parameters. (**a**) PEMSD7(M) data experimental results; (**b**) PEMSD7(L) data experimental results.

Since the number of stacked layers determines the model’s ability to capture features along the temporal dimension, and the number of hidden units controls the feature representation capacity of each layer, a series of experiments were conducted to explore the impact of hyperparameter settings on model performance. These experiments were performed on the PEMSD7(M) and PEMSD7(L) datasets with different network configurations. The study focused on two hyperparameters: the number of stacked layers L{2,3,4} and the number of hidden units Hidden_num{32,64,128}. The experimental results are shown in Figure 7. Analysis of the results reveals that the performance of the D-MGDCN-CLSTM model does not continuously improve with an increase in the number of stacked layers or hidden units. For the PEMSD7(M) dataset, the prediction performance stabilizes when using three-layer ConvLSTM with Hidden_num = 128. For the PEMSD7(L) dataset, the prediction performance stabilizes with two-layer ConvLSTM and Hidden_num = 128.

The number of DWT decomposition levels determines the granularity at which the model extracts spatio-temporal features from the raw traffic data. Too few decomposition levels may lead to insufficient feature extraction, while too many levels may introduce excessive detail or noise, negatively impacting model performance.

To select an appropriate decomposition level, experiments were conducted on two datasets, with the results shown in Table 3. The results of these experiments indicate that as the decomposition level increases, the model’s performance gradually stabilizes. Further increasing the decomposition level does not significantly improve performance. Considering the balance between prediction accuracy and model complexity, the decomposition level is set to 3 for the PEMSD7(M) dataset, and 4 for the PEMSD7(L) dataset, to achieve an optimal trade-off between performance and complexity.

The D-MGDCN-CLSTM model demonstrates strong generalization ability in capturing the spatio-temporal features of traffic networks, making it adaptable to different scales and types of traffic networks. It performs well in modeling both short-term dynamics and long-term dependencies. However, the model’s performance depends on the quality of the graph structure, with limited adaptability to anomalous events and sparse traffic networks, and it has relatively high computational resource requirements. Additionally, the complexity of tuning high-dimensional hyperparameters may increase the difficulty of deploying the model. To enhance the model’s practicality, the construction of adaptive graphs, the integration of external information, and a lightweight design could be considered to improve its generalization ability and address its potential limitations.

### 4.6. Ablation Experiment

In addition to verifying the impact of the proposed hyperparameters on the model, five additional comparison models were designed to gain a deeper understanding of the unique contributions of each module to the traffic prediction task. These comparison models were developed to comprehensively evaluate the methods introduced:

(1) MGDCN-CLSTM: Does not consider the impact of missing data.
(2) K-MGDCN-CLSTM: Fills missing data using the average of the top K nearest neighbors based on Euclidean distance.
(3) D-MGDCN-GRU: GRU is used to process the approximation coefficients.
(4) D-MGDCN-BiLSTM: BiLSTM is used to process the approximation coefficients.
(5) A^Dis^: Only considers the predefined adjacency matrix.
(6) A^P+S^: Only considers the similarity matrix.
(7) D-MGDCN: Utilizes only the MGDCN module to model detail coefficients, capturing the short-term fluctuation characteristics of traffic data.
(8) D-Conv-LSTM: Utilizes only the Conv-LSTM module to model approximation coefficients, capturing the long-term trend characteristics of traffic data.
(9) D-MGDCN-CLSTM: Combines all modules.

Table 4 shows the average prediction performance of different module combinations on the PeMSD7(M) and PeMSD7(L) datasets. The experimental results indicate that the MGCN-CLSTM model, which does not account for missing data imputation, performs poorly in predictions. This demonstrates that data completeness is crucial for the model’s ability to capture traffic patterns, understand the dynamic changes in traffic flow, and improve prediction accuracy. Compared to the similarity-based imputation method, the performance of the K-MGDCN-CLSTM model, which uses Euclidean distance to fill in missing data, is slightly worse. This suggests that the Euclidean distance-based imputation method is insufficient for capturing the inherent complexity and dynamic changes in spatio-temporal traffic features.

Additionally, to verify that Conv-LSTM can better capture long-term trends in time series, two models were designed using GRU and BiLSTM to process the approximation coefficients: D-MGDCN-GRU and D-MGDCN-BiLSTM. The experimental results show that, compared to GRU and BiLSTM, the D-MGDCN-ConvLSTM model, which uses Conv-LSTM, has a stronger ability to capture long-term trends in traffic time series. This further confirms that the Conv-LSTM structure is more effective in processing the approximation coefficients obtained by the DWT, thereby improving prediction accuracy and model stability.

At the same time, we observed that the performance of the models A^Dis^ (which only considers the predefined adjacency matrix) and A^P+S^ (which only considers the similarity matrix) is slightly inferior compared to the D-MGDCN-CLSTM model. This further confirms that combining both the predefined adjacency matrix and the similarity matrix not only accounts for geographic proximity and functional similarity, but also captures the nonlinear correlations in complex traffic networks. This combination provides more comprehensive contextual information for traffic flow prediction.

To validate the synergy between the MGDCN and Conv-LSTM, two variants were designed: D-MGDCN and D-Conv-LSTM. Experimental results show that the D-MGDCN-CLSTM model, which models both short-term fluctuations and long-term trends, outperforms the D-MGDCN, with MAE improvements of 2.4% and 2.5% and RMSE improvements of 2.6% and 0.9% on the two datasets, respectively. Compared to D-Conv-LSTM, it achieved MAE improvements of 3.1% and 3.6% and RMSE improvements of 2.9% and 1.4%. These results effectively demonstrate that the combination of MGDCN and Conv-LSTM leverages their complementary strengths, enhancing the model’s predictive performance.

**Figure 8:**
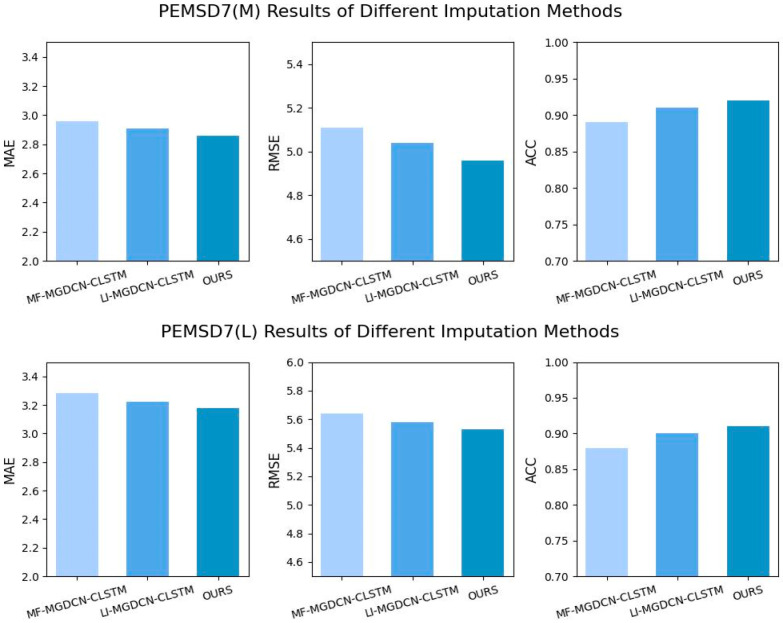
Comparison of experimental results for different interpolation methods.

To verify the advantages of DTW in handling missing data, two variants, LI-MGDCN-CLSTM and MF-MGDCN-CLSTM, were designed, using linear interpolation and mean interpolation, respectively, to fill in the missing data. The experimental results are shown in Figure 8. The results demonstrate that DTW minimizes the deviation between the filled data and the real data, whereas mean and linear interpolation methods result in poorer predictive performance, due to their failure to consider the temporal characteristics and dynamic trends of the data. This highlights that DTW dynamically matches local patterns in time series, preserving temporal dependencies and local trend features, while avoiding the over-smoothing or disruption of temporal structures that traditional interpolation methods may cause.

### 4.7. Visual Analysis

**Figure 9:**
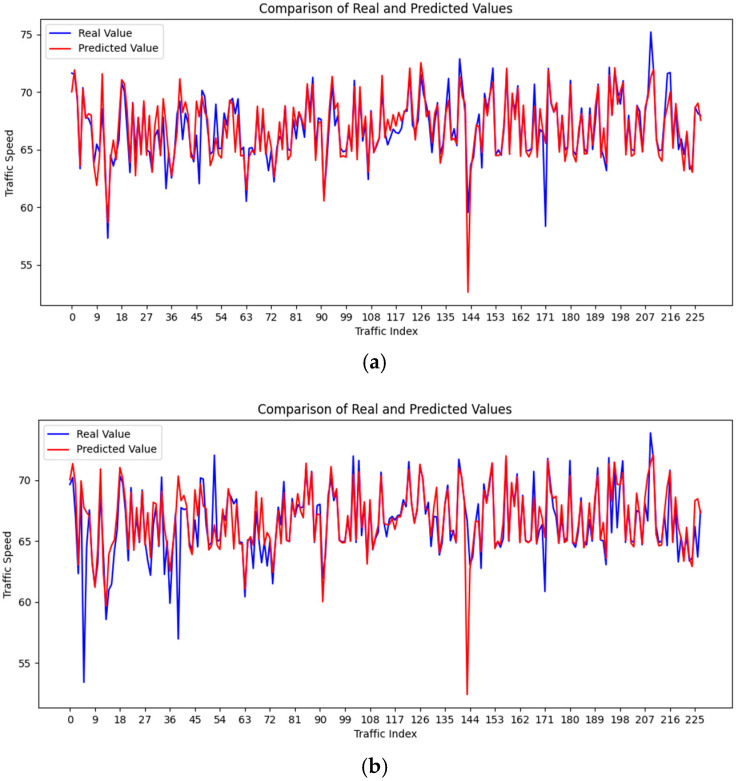

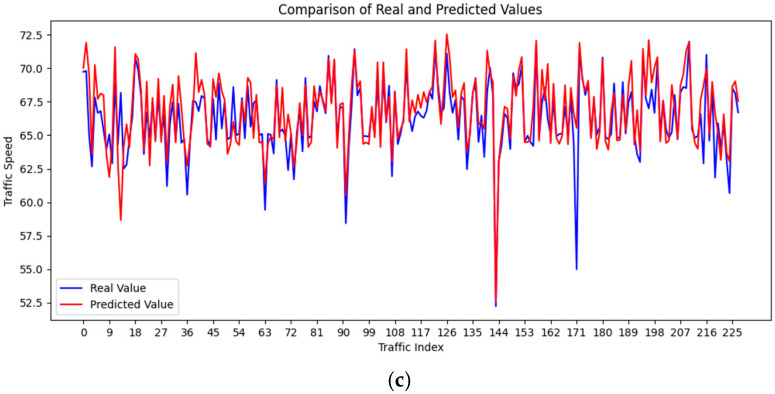
PEMSD7(M) prediction visualization. (**a**) Comparison chart for 15 min prediction; (**b**) Comparison chart for 30 min prediction; (**c**) Comparison chart for 60 min prediction.

To evaluate the practical performance of the proposed D-MGDCN-CLSTM model in traffic speed prediction, experiments were conducted on the PEMSD7(M) dataset for 15 min, 30 min, and 60 min forecasts. The comparison results are shown in Figure 9. The results indicate that the model’s predictions align well with the overall trend of actual traffic speed data across different time spans, particularly during periods of stable traffic flow, for which it accurately captures the dynamic changes in traffic speed. However, there are discrepancies between the predicted and actual values, especially during sudden traffic changes or irregular fluctuations, suggesting that the model struggles to fully predict abrupt variations in traffic speed.

Figure 9a presents the 15 min prediction results, showing that the model effectively captures short-term traffic flow changes. For most sensor nodes (e.g., nodes 0–100 and 180–225), the predicted values closely match the actual values, demonstrating the model’s high accuracy in capturing short-term fluctuations. However, for certain sensor nodes (e.g., around nodes 40–45, 17, and 207), where the traffic flow exhibits sharp variations, the predictions exhibit some lag, indicating a slight delay in the model’s response to extreme changes.

Figure 9b shows the 30 min prediction results. While the overall trend remains smooth, the predictions for most sensor nodes (e.g., nodes 0–100 and 180–220) align well with the actual traffic speed changes. Compared to the 15 min predictions, the error range increases, particularly during high-traffic fluctuation periods (e.g., nodes 120–140 and 160–180), for which underestimations and overestimations are evident, leading to reduced prediction accuracy. This suggests that the model’s ability to capture sudden changes and detailed fluctuations diminishes as the time span increases.

Figure 9c illustrates the 60 min prediction results. While the model captures the overall trend of traffic speed, the errors become significantly larger in regions with irregular fluctuations (e.g., nodes 120–140 and 160–180), alternating between underestimations and overestimations. Compared to the 15 min and 30 min predictions, the 60 min results reveal that the increasing time span exacerbates cumulative errors, causing the prediction curve to deviate further from the actual values. Overall, the model performs well in modeling long-term trends, but requires further optimization to improve its ability to handle irregular fluctuations.

From the experimental results of the visual analysis, it is evident that the D-MGDCN-CLSTM model achieves high prediction accuracy under normal traffic conditions, particularly excelling in short-term predictions. However, when sudden anomalous events occur in the traffic system (e.g., abrupt increases or decreases in traffic speed), the model’s error increases significantly. For instance, in the 30 min predictions, the impact of anomalous speed fluctuations becomes more pronounced, especially during periods of frequent peak traffic fluctuations (e.g., intervals 120–160 and 160–180), for which the model’s deviations are more prominent, struggling to quickly respond to abrupt traffic trend changes. In the 60 min predictions, the prediction deviations during anomalous periods are even more noticeable, and the model exhibits a further reduced ability to adapt to long-term anomalies. Overall, the model primarily relies on historical spatio-temporal patterns for predictions, and responds slowly to sudden and nonlinear dynamic changes, revealing its limitations in handling anomalous events. These limitations are particularly evident when both the prediction duration and the amplitude of traffic flow fluctuations increase. To address this issue, future model optimizations should focus on incorporating mechanisms for detecting and responding to anomalous events.

## 5. Conclusions

To address the negative impact of missing data and the failure of existing models to consider both short-term fluctuations and long-term trends, this paper proposes a traffic prediction model based on Multi-Graph Gated Dilated Convolution and Conv-LSTM (D-MGDCN-CLSTM) to predict future traffic conditions. The model incorporates the DTWN algorithm to effectively handle missing data, and different models are designed to process the various components derived from the DWT, considering both short-term fluctuations and long-term trends in traffic. To capture short-term fluctuations, a predefined adjacency matrix and a similarity matrix are designed to effectively account for the structure of the traffic network under different semantic meanings. These are combined with a gating mechanism and dilated convolution to capture temporal features. For accurately capturing long-term trends in the time series, a Conv-LSTM model is introduced. Extensive experiments and validations were conducted on two public datasets, PeMSD7(M) and PeMSD7(L), and the results show that the proposed D-MGDCN-CLSTM model achieves higher prediction accuracy.

Future research should expand on the current work in two directions. Firstly, evaluating the impact of different preprocessing methods for the DWT components on prediction performance: this includes investigating whether further smoothing is needed to emphasize long-term trends, assessing the contributions of approximation coefficients and detail coefficients to prediction performance, and exploring whether different weights can be assigned to these components. Secondly, assessing the influence of external environmental factors (e.g., weather conditions, points of interest (POI), holidays, and traffic accidents): this involves transforming these factors into features, incorporating them through feature embedding, or constructing multi-graph structures (e.g., semantic graphs based on POI and traffic accidents) to enhance the representation of complex dynamics. The goal is for the model to not only accurately predict regular traffic flow, but also effectively capture and respond to anomalous events and nonlinear changes. This would provide more effective decision-making support for intelligent traffic system management and urban planning.

## Figures and Tables

**Table 1:** Dataset details.

Dataset	Time Range	Time Type	Nodes	Time Steps	Time Interval
PEMSD7(M)	1 May 2012–20 June 2012	Weekday	228	12,672	5 min
PEMSD7(L)	1 May 2012–20 June 2012	Weekday	1026	12,672	5 min

**Table 2:** Experimental results of different models.

Models	PEMSD7(M)			PEMSD7(L)		
	MAE	RMSE	ACC	MAE	RMSE	ACC
GCN	8.41	8.43	0.80	11.86	11.87	0.80
T-GCN	7.66	10.89	0.87	7.87	11.31	0.76
STSGCN	4.12	7.74	0.82	8.48	14.51	0.81
STGCN	3.86	6.79	0.89	3.89	6.83	0.87
DCRNN	3.83	7.18	0.91	4.33	8.33	0.89
GraphWaveNet	3.19	6.24	0.90	3.75	7.09	0.90
ASTGCN	3.14	6.18	0.86	3.51	6.81	0.84
LSGCN	3.05	5.98	0.88	3.49	6.55	0.86
STGODE	2.97	5.66	0.90	3.42	5.98	0.88
STFGNN	2.90	5.79	0.91	3.22	5.91	0.90
D-MGDCN-CLSTM	2.86	4.96	0.92	3.18	5.53	0.91

**Table 3:** Comparison of experimental results for different decomposition levels.

Decomposition Levels	PEMSD7(M)			PEMSD7(L)		
	MAE	RMSE	ACC	MAE	RMSE	ACC
1	2.874	4.966	0.907	3.190	5.539	0.902
2	2.869	4.961	0.910	3.186	5.536	0.905
3	**2.863**	**4.958**	**0.915**	3.182	5.531	0.909
4	2.866	4.962	0.911	**3.178**	**5.527**	**0.912**
5	2.870	4.965	0.908	3.181	5.530	0.904

**Table 4:** Experimental results of different module combinations.

Models	PEMSD7(M)			PEMSD7(L)		
	MAE	RMSE	ACC	MAE	RMSE	ACC
MGDCN-CLSTM	11.53	18.41	0.67	14.61	20.29	0.54
K-MGDCCN-CLSTM	2.91	5.01	0.90	3.22	5.57	0.90
D-MGDCN-GRU	3.22	5.15	0.90	3.49	5.95	0.88
D-MGDCN-BiLSTM	2.93	5.04	0.90	3.34	5.84	0.90
A^Dis^	2.89	5.00	0.90	3.21	5.55	0.89
A^P+S^	2.91	5.02	0.89	3.23	5.56	0.88
D-MGDCN	2.93	5.09	0.89	3.26	5.58	0.89
Conv-LSTM	2.95	5.11	0.88	3.30	5.61	0.88
D-MGDCN-CLSTM	2.86	4.96	0.92	3.18	5.53	0.91

## Data Availability

Not available.

## References

[1] Zhang J., Wang F.Y., Wang K., Lin W.H., Xu X., Chen C. (2011). Data-Driven Intelligent Transportation Systems: A Survey. IEEE Trans. Intell. Transp. Syst..

[2] Williams B.M., Hoel L.A. (2003). Modeling and forecasting vehicular traffic flow as a seasonal ARIMA process: Theoretical basis and empirical results. J. Transp. Eng..

[3] Zivot E., Wang J. (2006). Vector autoregressive models for multivariate time series. Modeling Financial Time Series with S-PLUS®.

[4] Guo S., Lin Y., Wan H., Li X., Cong G. (2021). Learning dynamics and heterogeneity of spatial-temporal graph data for traffic forecasting. IEEE Trans. Knowl. Data Eng..

[5] Zhang W., Zhu K., Zhang S., Chen Q., Xu J. (2022). Dynamic graph convolutional networks based on spatiotemporal data embedding for traffic flow forecasting. Knowl.-Based Syst..

[6] Zheng Q., Zhang Y. (2023). TAGnn: Time Adjoint Graph Neural Network for Traffic Forecasting. International Conference on Database Systems for Advanced Applications.

[7] Li L., He S., Zhang J., Ran B. (2016). Short-term highway traffic flow prediction based on a hybrid strategy considering temporal–spatial information. J. Adv. Transp..

[8] Bae B., Kim H., Lim H., Liu Y., Han L.D., Freeze P.B. (2018). Missing data imputation for traffic flow speed using spatio-temporal cokriging. Transp. Res. Part C Emerg. Technol..

[9] Shepelev V., Slobodin I., Almetova Z., Nevolin D., Shvecov A. (2023). A hybrid traffic forecasting model for urban environments based on convolutional and recurrent neural networks. Transp. Res. Procedia.

[10] Méndez M., Merayo M.G., Núñez M. (2023). Long-term traffic flow forecasting using a hybrid CNN-BiLSTM model. Eng. Appl. Artif. Intell..

[11] Song C., Cao J., Zhao Q., Sun S., Xia W., Sun L. (2024). A high-precision crown control strategy for hot-rolled electric steel using theoretical model-guided BO-CNN-BiLSTM framework. Appl. Soft Comput..

[12] Kipf T.N., Welling M. (2016). Semi-Supervised Classification of graph convolutional networks with laplacian rank con-straints. Neural Process. Lett..

[13] Bai L., Yao L., Li C., Wang X., Wang C. (2020). Adaptive graph convolutional recurrent network for traffic forecasting. Adv. Neural Inf. Process. Syst..

[14] Lan S., Ma Y., Huang W., Wang W., Yang H., Li P. Dstagnn: Dynamic spatial-temporal aware graph neural network for traffic flow forecasting. Proceedings of the 39th International Conference on Machine Learning.

[15] Li M., Zhu Z. Spatial-Temporal Fusion Graph Neural Networks for Traffic Flow Forecasting. Proceedings of the 35th AAAI Conference on Artificial Intelligence (AAAI-21).

[16] Tian R., Wang C., Hu J., Ma Z. (2023). Multi-scale spatial-temporal aware transformer for traffic prediction. Inf. Sci..

[17] Khan S.I., Hoque A.S.M.L. (2020). SICE: An improved missing data imputation technique. J. Big Data.

[18] Andridge R.R., Little R.J.A. (2010). A review of hot deck imputation for survey non-response. Int. Stat. Rev..

[19] Qu L., Zhang Y., Hu J., Jia L., Li L. A BPCA based missing value imputing method for traffic flow volume data. Proceedings of the 2008 IEEE Intelligent Vehicles Symposium (IV).

[20] Liu Z., Sharma S., Datla S. (2008). Imputation of missing traffic data during holiday periods. Transp. Plan. Technol..

[21] Joelianto E., Fathurrahman M.F., Sutarto H.Y., Semanjski I., Putri A., Gautama S. (2022). Analysis of spatiotemporal data imputation methods for traffic flow data in urban networks. ISPRS Int. J. Geo-Inf..

[22] Zeng W., Wang K., Zhou J., Cheng R. (2023). Traffic Flow Prediction Based on Hybrid Deep Learning Models Considering Missing Data and Multiple Factors. Sustainability.

[23] Laña I., Olabarrieta I., Vélez M., Del Ser J. (2018). On the imputation of missing data for road traffic forecasting: New insights and novel techniques. Transp. Res. Part C Emerg. Technol..

[24] Laqueur H.S., Shev A.B., Kagawa R.M.C. (2021). SuperMICE: An ensemble machine learning approach to multiple imputation by chained equations. Am. J. Epidemiol..

[25] Song C., Lin Y., Guo S., Wan H. Spatial-temporal synchronous graph convolutional networks: A new framework for spatial-temporal network data forecasting. Proceedings of the 34th AAAI Conference on Artificial Intelligence (AAAI-20).

[26] Zhao L., Song Y., Zhang C., Liu Y., Wang P., Lin T., Deng M., Li H. (2019). T-gcn: A temporal graph convolutional network for traffic prediction. IEEE Trans. Intell. Transp. Syst..

[27] Yu B., Yin H., Zhu Z. (2018). Spatio-temporal graph convolutional networks: A deep learning framework for traffic forecasting. arXiv.

[28] Li Y., Yu R., Shahabi C., Liu Y. (2017). Diffusion convolutional recurrent neural network: Data-driven traffic forecasting. arXiv.

[29] Wu Z., Pan S., Long G., Jiang J., Zhang C. (2019). Graph wavenet for deep spatial-temporal graph modeling. arXiv.

[30] Guo S., Lin Y., Feng N., Song C., Wan H. Attention based spatial-temporal graph convolutional networks for traffic flow forecasting. Proceedings of the 33rd AAAI Conference on Artificial Intelligence.

[31] Huang R., Huang C., Liu Y., Dai G., Kong W. (2020). LSGCN: Long short-term traffic prediction with graph convolutional networks. IJCAI.

[32] Fang Z., Long Q., Song G., Xie K. Spatial-temporal graph ode networks for traffic flow forecasting. Proceedings of the 27th ACM SIGKDD Conference on Knowledge Discovery & Data Mining.

